# Descriptive Analysis of Adaptive Behavior in Phelan–McDermid Syndrome and Autism Spectrum Disorder

**DOI:** 10.3389/fnins.2022.893003

**Published:** 2022-07-04

**Authors:** Sergio Serrada-Tejeda, Rosa M. Martínez-Piédrola, Nuria Máximo-Bocanegra, Patricia Sánchez-Herrera-Baeza, Marta Pérez-de-Heredia-Torres

**Affiliations:** Department of Physical Therapy, Occupational Therapy, Rehabilitation and Physical Medicine, Rey Juan Carlos University, Madrid, Spain

**Keywords:** Phelan-McDermid syndrome, *SHANK3*, adaptive behavior, autism spectrum disorder, participation

## Abstract

**Introduction:**

The variety in symptomatology and clinical presentation of individuals diagnosed with Phelan-McDermid Syndrome (PMS) can delay medical diagnosis, so identifying specific neurobehavioral variables and facilitating differential diagnosis with patients with idiopathic Autism Spectrum Disorder (ASD) can guide early detection.

**Methods:**

A descriptive analysis of the level of adaptive behavior in 50 patients diagnosed with PMS was performed (*SHANK3*_deletion_: *N* = 44*; SHANK*_*mutation*_: *N* = 6). Subsequently, a comparative analysis was performed with 28 children aged between 4 years and 6 years and 11 months (*SHANK3*_deletion_ = 14; ASD = 14). Differences between the two groups were evaluated and Bonferroni correction was applied for multiple comparisons.

**Results:**

Differences were identified in the variables of communication (*z* = −2.715, *p* = 0.007), Self-Direction (*z* = −2.199, *p* = 0.028) and social participation (*z* = −3.190, *p* = 0.001), with better adaptive behavior skills being observed in participants with a SHANK3_*mutation*_. Better adaptive skills in the sample of participants with _*ASD*_, were found and statistically significant differences were identified in the variables of academic skills (*z* = −3.084, *p* = 0.002), use of community resources (*z* = −1.889, *p* = 0.050) and health and safety (*z* = −2.90, *p* = 0.004).

**Conclusion:**

Participants with *SHANK3*_*mutation*_ show better communication and social participation skills than those with a diagnosis of *SHANK3*_deletion_. The observed differences between ASD and individuals with PMS reflect deficits in practical and conceptual adaptive skills that may limit and hinder daily adaptive functioning.

## Introduction

Phelan-McDermid syndrome (PMS) is a genetic disorder and one of the most common single-gene forms associated with the diagnosis of autism spectrum disorder (ASD). The current classification system for PMS, differentiates between PMS-*SHANK3* individuals and PMS-*SHANK3* unrelated (Phelan et al., 2022). In PMS-*SHANK3* individuals the neurobehavioral phenotype is due to a deletion of the terminal end of chromosome 22 in the 22q13 region, unbalanced translocations, ring chromosomes, as well as a consequence of pathogenic variants or mutations of the *SHANK3* gene (Bonaglia et al., 2001; Luciani et al., 2003; Wilson et al., 2003). This genetic abnormality, responsible for most of the clinical features and signs (Bonaglia et al., 2001; Wilson et al., 2003; Delhaye et al., 2009), is of special interest due to the putative association between ASD and PMS as the *SHANK3* gene is one of the many genes implicated in the etiology of ASD (Durand et al., 2006; Bill and Geschwind, 2009; Uchino and Waga, 2013), and whose deficiency is associated with 0.5–2% of cases of ASD and intellectual disability (Soorya et al., 2013; Leblond et al., 2014; Richards et al., 2017).

Despite the increase in the diagnosis of PMS as a result of advanced molecular genetic testing (Phelan and McDermid, 2012), the first study conducted in Spain (Gómez Taylor et al., 2020) describes a very low prevalence (4 × 10^–4^/10,000), and whose figures are far from the estimated global rate, which is in the range of 2.5–10 per million births, although this is likely to be a gross underestimate as it is based on a limited statistical analysis (National Organization for Rare Disorders. Phelan-McDermid Syndrome). In this sense, and as in other cultural contexts (Boccuto et al., 2013; Zwanenburg et al., 2016), it can be considered an underdiagnosed disease, probably due to the non-specific characteristics and symptomatology of the diagnosis.

As in other genetic diseases, early detection and establishment of an initial diagnosis is a key element. In individuals with PMS, this assessment process, can often be hampered by the presence of associated impairments in cognitive ability, communication, or interaction with the environment (Phelan et al., 2011; Soorya et al., 2017). Most reported cases of PMS are caused by deletions in 22q13. 3 (Bonaglia et al., 2011; Soorya et al., 2013; Sarasua et al., 2014a), and, although information on individuals with mutation in SHANK3 is still rare (De Rubeis et al., 2018) and a small number of cases have been described (Soorya et al., 2013; Holder and Quach, 2016; De Rubeis et al., 2018), it has been estimated that haploinsufficiency in SHANK3 could account for about 1% of ASD cases (Betancur and Buxbaum, 2013; Leblond et al., 2014; De Rubeis et al., 2018). In recent studies, genetic analysis indicates that the size of the deletion and severity of clinical manifestations are positively correlated (Luciani et al., 2003; Wilson et al., 2003; Soorya et al., 2013; Sarasua et al., 2014a), with clinical signs such as hypotonia (Luciani et al., 2003; Wilson et al., 2003; Sarasua et al., 2011), developmental delay (Luciani et al., 2003; Wilson et al., 2003; Sarasua et al., 2011), dysmorphic features (Soorya et al., 2013), and language and communication difficulties (Sarasua et al., 2014b). However, other studies report that subjects with smaller deletions are also severely affected (Misceo et al., 2011; Sarasua et al., 2014b; Oberman et al., 2015), but show more favorable development (Zwanenburg et al., 2016; Cammarata-Scalisi et al., 2022).

Therefore, and due to the variety in symptomatology and clinical presentation, research has focused on elaborating detailed phenotypes of development, communication (Sarasua et al., 2014a) or behavioral variables (Phelan and McDermid, 2012; Richards et al., 2017; Droogmans et al., 2019) to facilitate the diagnosis of PMS. To date, although traits similar to those observed in ASD have been identified, such as poor eye contact, the presence of stereotyped movements or self-stimulation (Soorya et al., 2013; Droogmans et al., 2019), in some instances, individuals with PMS do not meet the criteria for a diagnosis of ASD. In addition, it has been observed that the difficulties identified in younger individuals are often associated with emotional and behavioral difficulties, as well as a greater presence of ASD traits and deficits in social communication (Oberman et al., 2015), which may negatively impact adaptive skills that are necessary for daily functioning (Shaw et al., 2011; Reierson et al., 2017; Droogmans et al., 2019).

Therefore, and due to the scarce information and studies with individuals with PMS-*SHANK3*, the objectives of the present study were to obtain detailed information on the adaptive behavioral profile of individuals depending on the genetic alteration presented (PMS_deletion_ vs. PMS_*mutation*_) and to analyze whether there are significant differences compared to a matched sample of participants with ASD.

## Materials and Methods

### Study Design

The data from this descriptive study are part of a larger longitudinal research project examining the evolution of adaptive behavior and sensory difficulties in the PMS population. The study was approved by the Clinical Research Ethics Committee of the Universidad Rey Juan Carlos. The families of the study participants completed the informed consent document and accepted the sending of supporting documentation for diagnostic confirmation.

This study was conducted in Spain and the collection, management, storage, communication, and transfer of all data was completed in accordance with the provisions of the Declaration of Helsinki World Medical Association [WMA], 2017, the data protection law in force in the General Data Protection (EU Regulation 2016-679 of the European Parliament and of the Council of 27 April 2016), and current Spanish regulations on personal data protection.

### Participants

Convenience sampling was performed between July 2020 and December 2020. The participant samples of the present study are constituted by two groups: a first group of participants with a diagnosis of PMS and a second group with a diagnosis of ASD. For both participant samples the parents signed and accepted the informed consent form. Participants with PMS were recruited through an internal communication sent by the management of the Asociación Síndrome Phelan-McDermid (Spain). The participants with a diagnosis of ASD were recruited through a public early care service of the Community of Madrid that attends to children with special needs, transitory or permanent, caused by deficiencies, alterations in development or risk of suffering them, and who have been previously evaluated by the Regional Center for Coordination and Evaluation of Children (CRECOVI) of the Community of Madrid.

PMS participants met the inclusion criteria if they had a confirmed diagnosis by demonstration of a *SHANK3*_deletion_ with comparative genomic hybridization (CGH) or by whole exome sequencing (WES) in case of a *SHANK3*_mutation_. Participants with ASD were included if they were within the age range and had a confirmed diagnosis of ASD identified by a physician, psychologist, neurologist, or psychiatrist, as described in the DSM-5 American Psychiatric Association [APA], 2013. ASD participants had no associated medical diagnoses, so no genetic alterations were reported in any of them. To perform the comparative analysis, an age-matched-subsample of the SHANK3_deletion_ group was included and compared with a group of children with a diagnosis of ASD and an age of less than 6 years and 11 months.

### Procedure

To complete the ABAS-II questionnaire, a copy was sent to the parents and/or legal guardians of each participant for completion. Before beginning the completion of the questionnaire, families were instructed on the importance of understanding the general instructions and the specific criteria for scoring. In this way, the research team ensured that all participants’ families had the same information to assign the score for the frequency of the behaviors. However, when necessary, the research team contacted by telephone those families who requested help in completing the questionnaire due to difficulties in understanding the instructions, or if some of the questionnaire items remained unanswered. In both situations, the researchers clarified doubts in the completion of the questionnaire and made sure that the families assigned a specific value for all the items in the questionnaire.

A separate document was also sent to collect information related to the patients, including sociodemographic data, type of genetic disorder, as well as attendance to specific rehabilitation treatments.

### Outcome Assessments

The Adaptive Behavior Assessment Questionnaire (ABAS-II): provides a comprehensive assessment of the adaptive skills of individuals from birth to 89 years of age (Montero and Fernández-Pinto, 2013). The ABAS-II is a very valuable tool for the assessment of individuals who may have difficulties with the daily adaptive skills necessary for effective functioning in different environments. The ABAS-II assesses ten specific areas of adaptive skills that are grouped into three indexes or domains of adaptive behavior. Both the adaptive skill areas and domains are based on the American Association on Intellectual Developmental Disabilities (AAIDD) definition of adaptive behavior. The ABAS-II provides a General Adaptive Composite (GAC) that summarizes performance in all adaptive skill areas: Conceptual Domain (Communication, Functional (pre)academic skills, and Self-Direction); Social Domain (Leisure and social interaction skills); and Practical Domain (Use of community resources, Home or school life, Health and safety, Self-care, Motor skills, and Employment). Composite scores for Conceptual, Social and Practical domains, as well as the GAC, have a score of 100 and a standard deviation of 15, and in case of the adaptive skill areas, the raw scores are converted to scaled scores with a mean of 10 and a standard deviation of 3. Its psychometric properties demonstrated high test-retest reliability (*r* > 0.80) and adequate validity and internal consistency (GAC: *r* > 0.90; conceptual, social, and practical domains: *r* > 0.83).

### Statistical Analysis

Basic descriptive methods were used for the statistical analysis of the sample. For qualitative variables, the number of cases present in each category and the corresponding percentage were calculated and, for quantitative variables, the median and interquartile range were calculated as they did not follow a normal distribution.

Due to the difference in sample size between PMS subsamples, a descriptive analysis of the results obtained was performed. Moreover, for a comparative analysis the Wilcoxon rank-sum test was used for participants comparisons (PMS_deletion_ vs ASD) and medians and quartiles were provided for each group. Estimates for the median of the treatment differences and Hodges-Lehmann 2-tailed 95% confidence intervals were provided for each group comparison. To correct for type I error Bonferroni correction was applied to adjust for multiple comparisons at a level of significance of *p* < 0.0035 (0.05/14). Statistical analysis was performed with the SPSS 25.0 program for Windows (Copyright© 2013 IBM SPSS Corp.). Differences considered statistically significant are those whose *p* < 0.05.

## Results

The final study sample consisted of a total of 50 participants with a diagnosis of PMS (*SHANK3*_deletion_: *N* = 44 and *SHANK3*_mutation_: *N* = 6) and a sample of 14 participants with a diagnosis of idiopathic ASD. The sociodemographic data of the samples are described in Table 1.

**TABLE 1:** Participants demographic data.

	Phelan-McDermid Syndrome				Autism Spectrum Disorder	
	*SHANK3* deletion *n* = 44		*SHANK3* mutation *n* = 6		*n* = 14	
**Overall age, mean** **(SD) Age group, mean (SD)/n** **(%)** 3–6 years 7–12 years 13–17 years 18–24 years 25–30 years 30 years or more	11.16 (7.91) 4.71 (0.61)/15 (34.1%) 8.94 (0.44)/16 (36.4%) 14.83 (0.70)/6 (13.6%) 21.00 (0.58)/3 (6.8%) 24.00 (2.00)/2 (4.5%) 35.50 (1.50)/2 (4.5%)		13.33 (0.51) 5.00 (0.00)/1 (16.7%) 8.68 (0.33)/3 (50%) - 18.00 (0.00)/2 (33.3%) –		4.71 (0.61) 4.71 (0.61)/14 (100%) – – – – –	
**Gender, N (%)** Male Female	22 (50%) 22 (50%)		2 (33.3%) 4 (66.7%)		11 (78.6%) 3 (2.4%)	
**Genetic defect, N (%)** Deletion Deletion size interval Mutation	44 (90%) 52 kb–8.53 Mb 6 (10%)		– – –		– – –	
**Caregiver, N (%)** Mother Father Both Another person	24 (54.5%) 1 (2.3%) 18 (40.9%) 1 (2.3%)		5 (83.3%) – 1 (16.7%) –		6 (42,9%) 1 (7.1%) 7 (50%) –	

**Treatment services, N (%)**	**Yes**	**No**	**Yes**	**No**	**Yes**	**No**

Physiotherapy	22 (50%)	22 (50%)	3 (50%)	3 (50%)	–	14 (100%)
Speech therapy	35 (79.5%)	9 (20.5%)	5 (83.3%)	1 (16.7%)	10 (71.2%)	4 (28.6%)
Psychotherapy	15 (34.1%)	29 (65.9%)	4 (66.7%)	2 (33.3%)	3 (21.4%)	11 (78.6%)
Occupational therapy	9 (20.5%)	35 (79.5%)	2 (33.3%)	4 (66.7%)	10 (71.4%)	4 (28.6%)
**Place of residence**						
Spain *Andalucía* *Madrid* *Valencia* *País Vasco* *Cataluña* *Navarra* *Castilla-La Mancha* *Murcia* *Asturias* *Islas Baleares* *Galicia* *Castilla León*	47 (87.2%) 9 (18.5%) 8 (16.7%) 4 (7.4%) 3 (5.6%) 5 (9.3%) 3 (5.6%) 3 (5.6%) 2 (3.7%) 2 (3.7%) 2 (3.7%) 2 (3.7%) 1 (1.9%)				– 14 (100%) – – – – – – – – –	
Italy Argentina	1 (1.9%) 2 (3.7%)				- –	

Table 2 shows the descriptive analysis between the subsamples of participants with PMS. The ranges of scores obtained by individuals with *SHANK3*_mutation_ showed unequal score values. In the adaptive skills of self-direction, leisure, social interaction, and home or school living, individuals with mutation showed adaptive skill values comprised within the expected mean performance. In comparison to individuals with deletion, adaptive skills scores showed little variability and values associated with very low performance.

**TABLE 2:** ABAS-II descriptive results according to PMS genetic defect.

	PMS	
	SHANK3_mutation_	SHANK3_deletion_
**General Adaptive Composite, median (IQR)**	53.00(51.00–71.50)	53.00(51.00–53.00)
**Conceptual, median (IQR)** Communication, median (IQR) Functional Academics, median (IQR) Self-Direction, median (IQR)	54.00(53.75–64.50) 1.00(1.00–3.25) 1.00 (1.00–1.00) 1.00 (1.00–7.25)	54.00(53.00–54.00) 1.00 (1.00–1.00) 1.00 (1.00–1.00) 1.00 (1.00–1.00)
**Social, median (IQR)** Leisure skills, median (IQR) Social interaction skills, median (IQR)	56.00(52.00–89.00) 1.00 (1.00–7.25) 3.00 (1.00–9.00)	53.00(51.00–53.00) 1.00 (1.00–1.00) 1.00 (1.00–1.00)
**Practical, median (IQR)** Self-Care, median (IQR) Home or School Living, median (IQR) Community Use, median (IQR) Health and Safety, median (IQR)	53.50(51.00–60.75) 1.00 (1.00–1.25) 3.00 (1.00–8.25) 1.00 (1.00–2.50) 1.00 (1.00–1.50)	56.00(53.00–56.00) 1.00 (1.00–1.00) 1.00 (1.00–5.00) 1.00 (1.00–1.00) 1.00 (1.00–1.00)
Motor, median (IQR)	1.00 (1.00–1.00)	1.00 (1.00–1.00)

*IQR, interquartile range; Atypical on ABAS-II adaptive skills subscales is ≤ 7. Atypical on ABAS-II GAC/Conceptual/Social/Practical scores is ≤ 85.*

In the adaptive behavior domains, similar results were obtained in both subsamples, with scores associated with very low levels of adaptive behavior. However, participants with a *SHANK3* mutation showed better adaptive behavior skills in the social domain, with scores in the middle range of performance.

The comparative analysis of participants with *SHANK3*_deletion_ and ASD is shown in Table 3. In this case, the age range was adjusted to ensure greater stability in the measurements. The resulting sample consisted of a total of 28 participants (*SHANK3*_deletion_ = 14 and ASD = 14) ranging in age from 4 years to 6 years and 11 months. The mean age for both samples (*SHANK3*_deletion_ and ASD) was 4.71 (0.61) Gender distribution of participants was 10 males and 4 females for the SHANK3_deletion_ group, and 11 males and 3 females for the ASD sample.

**TABLE 3:** Descriptive statistics and comparative analysis of the ABAS-II in PMS and ASD population.

	Diagnosis		Difference PMS_deletion_ – ASD		
	PMS	ASD	Estimated media	95% CI	*P*-value
General Adaptive Composite, median (IQR)	53.00 (53.00–53.75)	54.50 (53.00–61.75)	0.000	[0.00, 0.00]	0.179
Conceptual, median (IQR) Communication, median (IQR) Functional Academics, median (IQR) Self-Direction, median (IQR)	53.00 (53.00–54.75) 1.00 (1.00–1.00) 1.00 (1.00–1.00) 1.00 (1.00–2.50)	58.00 (53.00–60.50) 1.00 (1.00–1.50) 4.00 (1.00–5.50) 1.00 (1.00–3.50)	−3.00 0.00 −3.00 0.00	[−7.00, 0.00] [−0.00, 0.00] [−4.00, 0.00] [0.00, 0.00]	0.070 0.345 0.002* 0.471
Social, median (IQR) Leisure skills, median (IQR) Social interaction skills, median (IQR)	53.00 (53.00–57.25) 1.00 (1.00–2.50) 1.00 (1.00–1.00)	53.00 (53.00–63.50) 1.00 (1.00–2.00) 1.00 (1.00–1.00)	0.00 0.00 0.00	[0.00, 0.00] [0.00, 0.00] [0.00, 0.00]	0.928 0.823 0.949
Practical, median (IQR) Self-Care, median (IQR) Home or School Living, median (IQR) Community Use, median (IQR) Health and Safety, median (IQR)	56.00 (56.00–56.00) 1.00 (1.00–1.00) 1.00 (1.00–5.25) 1.00 (1.00–1.00) 1.00 (1.00–1.00)	59.00 (56.00–72.00) 1.00 (1.00–6.50) 1.00 (1.00–6.25) 1.00 (1.00–6.00) 3.00 (1.00–6.50)	0.00 0.00 0.00 0.00 −2.00	[−8.00, 0.00] [0.00, 0.00] [−2.00, 0.00] [−1.00, 0.00] [−3.00, 0.00]	0.076 0.215 0.360 0.059 0.003*
Motor, median (IQR)	1.00 (1.00–1.00)	1.00 (1.00–2.00)	0.000	[0.00, 0.00]	0.062

*PMS, Phelan McDermid Syndrome; ASD, Autism Spectrum Disorder; CI, confidence interval; IQR, interquartile range. Atypical on ABAS-II adaptive skills subscales is ≤ 7.*

Atypical on ABAS-II GAC/Conceptual/Social/Practical scores is ≤ 85. The median differences between the treatment groups and 95% CIs were estimated with the Hodges-Lehmann method. P-value is from the Wilcoxon rank-sum test. *Groups differences if p < 0.05.

The estimate of the median of the *Functional Academics* skills differences between *SHANK3*_deletion_ and ASD was −3.00%. This adaptive behavior skill difference was statistically significant (*p* = 0.002). The estimate of the median of the *Health and Safety* skills differences between *SHANK3*_deletion_ and ASD was −2.00%. This adaptive behavior skills difference was statistically significant (*p* = 0.003). After Bonferroni correction, Functional Academics and Health and Safety were the only variables that showed significant differences between participants.

The rest of the comparative analysis of the participants with SHANK3 deletion and ASD showed that no statistically significant differences were found in the remaining adaptive skills. Similarly, neither in the general index (GAC) nor in the indexes of conceptual, social and practical adaptive skills showed differences between the two groups.

## Discussion

Identifying the developmental profile of adaptive behavior in a relatively large sample of people diagnosed with PMS in the Spanish population provides more detailed information on the characteristics of adaptive behavior, which may facilitate the identification of differential aspects during the evaluation and diagnosis process.

The results obtained in this study show as in previous studies (Havens et al., 2004; Dhar et al., 2010; Phelan et al., 2011; Shaw et al., 2011; Denayer et al., 2012; Phelan and McDermid, 2012; Zwanenburg et al., 2016; Brignell et al., 2021), that people with PMS present a low level of adaptive behavior, which limits effective participation in the environment and daily context. These results are similar to those obtained by Droogmans et al. (2019), in which they determine that people with PMS require a high need for assistance to perform daily activities.

The comparative analysis of the sample of participants with PMS, identified significant differences in the variables of communication, social participation, and self-direction among participants with PMS, with better skills observed in participants presenting a *SHANK3* gene mutation. As in the previous study, communication skills are severely affected with greater difficulties predominating in the ability to express language (De Rubeis et al., 2018). However, although communication delay is evident, individuals with a diagnosis of PMS and *SHANK3* mutation, appear to preserve better language skills, vs. those individuals affected by a *SHANK3* deletion (Soorya et al., 2013).

Although in our study, the size variability of genetic alterations was not determined, in the study by Soorya et al. (2013), it was observed that larger deletions were associated with greater difficulties in communication, whereas individuals with smaller deletions or point mutations showed characteristics similar to those of children with ASD. Similar results (Xu et al., 2020) support the effect that 22q13 gene deletions and *SHANK3* point mutations have on various clinical manifestations, such as developmental delay and language impairment.

Due to the similarities in the symptomatology of PMS and ASD, and similar to our study, current research has not identified significant differences in communicative or social areas. In the study developed by Richards et al. (2017), it was identified that the PMS sample showed greater difficulties in social reciprocity in showing and directing attention, observing, in addition, better skills in showing interest and responding to other people, and a lower tendency to show ritualistic and repetitive behaviors than in subjects with ASD. Similarly, Burdeus-Olavarrieta et al. (2021) identified that there were no differences between receptive and expressive communication skills, although the results showed a wide variability both within and between subjects.

Interestingly, previous studies indicate that the contributing factor to the clinical diagnosis of ASD was the social adaptive subdomain (Oberman et al., 2015) and that specific genetic deficits, but not total loss of genetic material, may explain ASD symptomatology in PMS. In other genetic syndromes with similar social adaptive profiles, such as fragile X syndrome, although an aberrant and maladaptive social behavior profile has been identified (Kaufmann et al., 2004), communication difficulties do not appear to be a differentiating feature vs. PMS (Richards et al., 2017). Likewise, in Down syndrome it has been identified that those with ASD symptomatology show more disruptive adaptive behavior and a greater presence of stereotypies and social withdrawal (Carter et al., 2007). Furthermore, although on the communicative aspects, language regression is a trait that has not been fully defined in PMS, it has been identified in previous studies (Macedoni-Lukšič et al., 2013; Figura et al., 2014; Cochoy et al., 2015). Unlike Rett syndrome, where regression occurs between the first and third year of life, in PMS the age of onset appears to be undefined (Bonaglia et al., 2011; Soorya et al., 2013).

In our study, two areas of adaptive skills were identified that differentiated the PMS sample from the ASD sample. In the case of the sample with PMS_deletion_, the scores associated with *Functional Academics* and *Health and Safety* skills showed no variability, and the values remained constant, showing limitations in the ability to participate actively and unsupervised in most of the skills expected for their age. Parents report that for all items assessed, their children are not able to engage in the behaviors described. In contrast, in the sample of participants with ASD, greater variability in scores was observed. Although the level of adaptive skills remains atypical (ABAS_subscales_ ≤ 7), they showed better scores in items related to prewriting skills, pre-reading and pre-mathematical concepts such as counting or sorting. In the case of safety and health skills, participants with ASD show needs for adult supervision in open spaces, and better behaviors in situations where adults give orders to avoid a dangerous situation.

In similar studies, such as the one developed by Shaw et al. (2011), most of the sample with PMS identified maladaptive behaviors related to difficulties in generating non-disruptive internalizing and externalizing behaviors. These behaviors can impact daily functioning and the skills needed to identify risk situations, requiring a higher level of assistance from the adult in order to perform daily activities effectively. However, although differences in score ranges were observed between the PMS subsamples and individuals with ASD, the differences were not statistically significant.

As a consequence of the difficulties shown in daily functioning and adaptive behavior, the needs generated by people with PMS in daily activities and routines must be addressed (Durand et al., 2006; Shaw et al., 2011; Denayer et al., 2012; Brignell et al., 2021). Although the scientific evidence on the speech, physical, occupational, and behavioral treatment of PMS is limited, having descriptive information on the developmental difficulties of individuals with PMS can facilitate and assist the intervention process of professionals, allowing them to improve the quality of their care, and address observable developmental difficulties more precisely.

## Limitations

Due to the social and health crisis caused by COVID-19 and the geographic distribution of the participants, it was not possible to assess aspects of development for which the patient’s presence is required, such as IQ. However, although social restrictions were imposed for the duration of the study, the self-administered assessment instruments were conducted and completed by the primary caregivers. Nevertheless, although the ABAS-II has been previously validated in Spain and has reliable psychometric properties, as it is a self-questionnaire, the possibility of some measurement bias should be considered. However, for future lines of research we have proposed to evaluate these areas, which are relevant in this population.

The sample size of the PMS subsamples is not matched. However, the subdivision has been performed in order to follow a similar methodology to studies conducted in the PMS population. Although the difference in sample sizes of the study is high, it was considered appropriate to include the information obtained in the *SHANK3*_*mutation*_ subsample, due to the limited number of studies reporting on its phenotype (Soorya et al., 2013; Holder and Quach, 2016; De Rubeis et al., 2018). However, although the comparative sample of individuals with SHANK3 deletion and ASD is age-matched, it was not possible to perform this same matching by gender, which could introduce additional confounding bias. However, the final ratio of males to females was close between the two groups. In addition, although it is specified that the participants with ASD have a diagnosis confirmed by a physician, none of them underwent a specific genetic analysis during the study.

## Conclusion and Future Directions

The described profiles indicate that genetic alterations in the diagnosis of PMS have a negative impact on the development and acquisition of adaptive skills, hindering effective participation in the context. This study expands information on an underdiagnosed pathology and determines quantifiable differences between PMS-*SHANK3* individuals, associating better communication and social participation skills in subjects with a genetic mutation. Due to average variability in sample sizes, the results should be analyzed carefully, as they may not be extrapolated to the general PMS-*SHANK3* population. However, due to the limited descriptions of adaptive profiles in both *SHANK3*_deletion_ and SHANK3_*mutation*_ individuals, these results may help to delineate their phenotypic profiles. The differences observed between children with ASD and with *SHANK3*_deletion_ identified greater deficits in practical and conceptual adaptive skills, which may negatively impact daily management by primary caregivers. However, these results cannot be extrapolated to the remaining PMS-*SHANK3* individuals. Future lines of research should address these difficulties and implementing therapeutic interventions aimed at facilitating and promoting their development should be essential to support the development of this population.

## Data Availability Statement

The raw data supporting the conclusions of this article will be made available by the authors, without undue reservation.

## Ethics Statement

The studies involving human participants were reviewed and approved by Comité de ética de la investigación de la Universidad Rey Juan Carlos. Written informed consent to participate in this study was provided by the participants’ legal guardian/next of kin.

## Author Contributions

SS-T and MP-H-T conceived the idea. SS-T, RM-P, and PS-H-B drafted the first version of the manuscript. SS-T was responsible for data storage and statistical analysis. MP-H-T and NM-B supervised the manuscript and reviewed the manuscript for intellectual content. All authors have read and agreed to the published version of the manuscript.

## Conflict of Interest

The authors declare that the research was conducted in the absence of any commercial or financial relationships that could be construed as a potential conflict of interest.

## Publisher’s Note

All claims expressed in this article are solely those of the authors and do not necessarily represent those of their affiliated organizations, or those of the publisher, the editors and the reviewers. Any product that may be evaluated in this article, or claim that may be made by its manufacturer, is not guaranteed or endorsed by the publisher.
